# Immunization with an mRNA DTP vaccine protects against pertussis in rats

**DOI:** 10.1128/iai.00520-23

**Published:** 2024-07-17

**Authors:** Graham J. Bitzer, Nicholas A. Fitzgerald, Megan A. DeJong, Casey Cunningham, Joshua A. Chapman, Dylan T. Boehm, Gage M. Pyles, Annalisa B. Huckaby, Sarah J. Miller, Spencer R. Dublin, Matthew D. Warden, Mariette Barbier, F. Heath Damron

**Affiliations:** 1 Department of Microbiology, Immunology, and Cell Biology, West Virginia University, Morgantown, West Virginia, USA; 2 West Virginia University Vaccine Development Center, West Virginia University, Morgantown, West Virginia, USA; University of California San Diego School of Medicine Department of Pediatrics, La Jolla, California, USA

**Keywords:** *Bordetella pertussis*, pertussis, whooping cough, leukocytosis, pertussis rat model, aerosol bacterial challenge, coughing

## Abstract

*Bordetella pertussis* is a Gram-negative bacterium that is the causative agent of the respiratory disease known as pertussis. Since the switch to the acellular vaccines of DTaP and Tap, pertussis cases in the US have risen and cyclically fallen. We have observed that mRNA pertussis vaccines are immunogenic and protective in mice. Here, we further evaluated the pertussis toxoid mRNA antigen and refined the formulation based on optimal pertussis toxin neutralization *in vivo*. We next evaluated the mRNA pertussis vaccine in Sprague-Dawley rats using an aerosol *B. pertussis* challenge model paired with whole-body plethysmography to monitor coughing and respiratory function. Female Sprague-Dawley rats were primed and boosted with either commercially available vaccines (DTaP or wP-DTP), an mRNA-DTP vaccine, or mock-vaccinated. The mRNA-DTP vaccine was immunogenic in rats and induced antigen-specific IgG antibodies comparable to DTaP. Rats were then aerosol challenged with a streptomycin-resistant emerging clinical isolate D420Sm1. Bacterial burden was assessed at days 1 and 9 post-challenge, and the mRNA vaccine reduced burden equal to both DTaP and wP-DTP. Whole-body plethysmography revealed that mRNA-DTP vaccinated rats were well protected against coughing which was comparable to the non-challenged group. These data suggest that an mRNA-DTP vaccine is immunogenic in rats and provides protection against aerosolized *B. pertussis* challenge in Sprague-Dawley rats.

## INTRODUCTION

Pertussis, also known as whooping cough, is a respiratory disease caused by the Gram-negative bacterium *Bordetella pertussis* (*Bp*). Recorded cases of pertussis in the US once were in the 100,000 s before the introduction of the whole-cell pertussis vaccine (wP; wP-DTP; DTP) in the 1940s (1). In tandem with reported case numbers, the mortality rate for infants before the introduction of the wP vaccine was 1,040 deaths/million population in 1934–1949 (2, 3). Further evaluation of mortality rates during the wP era suggested that if the wP vaccine was not introduced, infant deaths from pertussis would be between 4,000 and 8,000 deaths/year during the period of 1970–1974, but instead, a total of 52 deaths were recorded in the US during this period (3). Not only did the wP vaccine reduce infant mortality, but it also seemingly put an end to the cyclical endemics that pertussis would cause and during the height of wP usage case numbers were in the low 1,000’s per year (1, 4, 5). However, the wP was highly reactogenic and, due to safety concerns, was replaced by an acellular pertussis(aP) vaccine in the US (6, 7). Correlating with the introduction of the aP vaccine, case numbers within the US have risen to levels not observed since the 1970s. More recently, these levels have started to decrease from an average of 18,387 cases/year during the five years of 2015–2019 to an average of 4,120 cases/year during 2020 and 2021 (8). However, this overlaps with the SARS-CoV-2 pandemic (COVID-19) which sequestered the population in isolation. *Bp* is transmitted through respiratory droplets which would be trapped due to mask usage (9, 10). This potentially could be why there has been a decrease in pertussis cases, and if this is the case, we speculate that pertussis case numbers will increase again as social isolation and mask usage ends. As of mid-2024, the Centers for Disease Control and Prevention(CDC) has announced 4,876 cases which is far above COVID-19 pandemic levels.

Current aP vaccines (DTaP and Tdap and combo vaccines) induce a Th2 skewed immune responses, whereas wP vaccination and naïve infection induce a Th1/Th17 skewed immune response (11 – 14). This difference has been largely correlated to the increase of cases and has been a major focus of the field for two decades. The alum adjuvanted aP formulations drive a strong humoral response which does well at limiting disease severity but fails to clear bacterial carriage (15). Warfel et al. showed that aP immunization limits disease manifestations but does not limit asymptomatic carriage and transmission in non-human primates (16). On the other hand, wP immunization drives a Th1 skewed cell-mediated response (most likely due to large amounts of endotoxin in the vaccine) which was shown to clear *Bp* infections sooner than aP immunization in non-human primates (16). We hypothesize that a next-generation pertussis vaccine should have similar immune responses generated by wP vaccination and naïve convalescent immunity which have shown superior bacterial clearance than aP vaccination (11, 13, 14, 17, 18).

One next-generation option that we are evaluating is the mRNA vaccine platform for combo DTP vaccines. While this technology is not new, the use of mRNA vaccines against SARS-CoV-2 showed firsthand how beneficial this type of vaccine could be in our future fights against pathogenic microbes (19 – 21). mRNA vaccine technology allows for the development of vaccines that have the safety profile of aP formulations paired with the immune response and effectiveness of wP formulations. For instance, aP formulations typically contain between three and five antigens, whereas wP formulations have possibly included thousands of antigens (22). An mRNA vaccine can contain multiple antigens that include targets that are hard to purify and detoxify in traditional protein vaccines. Another benefit is the activation of toll-like receptor 7/8 by naked mRNA which induces IFN-γ production and skews toward a Th1 response matching more closely the response observed in wP immunization (23). In this study, we utilize an aerosol-challenged rat model to evaluate a 10-antigen mRNA vaccine encoding diphtheria, tetanus, and pertussis antigens. We have observed these mRNA formulations are immunogenic and protect mice from *Bp* experimental challenge.

Many different animal models have been used during the past century to study *Bp* pathogenesis and vaccine efficacy. Mice were the original animal model used to determine wP vaccine efficacy between different lots of produced vaccines in an intracranial (IC) challenge model known as the Kendrick test (24, 25). However, this is not a natural or normal route of *Bp* infection, so the intranasal (IN) instillation method was established to study *Bp* pathogenesis (26). The benefit of this challenging method with mice was that the deposition of bacteria in the respiratory system allowed for the assessment of bacterial burden and pertussis disease manifestations like leukocytosis (26, 27). However, mice that have been IN challenged do not exhibit an audible cough which is noted in human pertussis cases. In contrast, Wistar and Sprague-Dawley rats develop an audible cough upon intrabronchial (IB) and IN (28 – 31) challenge. The Sprague-Dawley rats IB challenged exhibited disease manifestations that were very similar to human pertussis manifestations including lung inflammation, bacterial burden in the lung, coughing, and hypoglycemia (31 – 33). Previously, we have used the coughing rat model to study coughing, as well as, protection by muscular, nasal, and oral administered pertussis vaccines (34, 35).

In our previous coughing rat model studies, we used (IN) administration of 10^8^ CFUs in 100 µL, 50 µL per nostril (34, 35). This model revealed robust coughing; however, we have appreciated that aerosol delivery causes different distribution of bacteria in the respiratory tract as well as less severe disease manifestations like leukocytosis and cytokine production (36). Aerosol delivery is widely used in mice, and thus, we aimed to adapt aerosol delivery to the coughing rat model (18, 37 – 42). Hypothetically, the aerosol challenge model mimics a more natural route of administration of bacteria than IN, IB, or IC instillation (36, 42). One advantage of the aerosol model is that it induces less severe disease manifestations in naïve animals (36). Bacterial burden is also affected by the aerosol challenge model. Fewer bacteria are instilled in the nares than from IN challenge with the same challenge dose concentration (36). The bacteria found in the nares could be over-represented in an IN challenge where such an unnaturally large bolus of bacteria is administered (36). In the case of aerosol challenge, we are better able to observe the effect of vaccine-mediated immunity with reduced initial deposit of bacteria.

Here, we developed a *Bp* isolate D420Sm1 which has only one single nucleotide polymorphism (SNP) from the original D420 isolate in the *rpsL* gene causing streptomycin resistance. The D420 isolate is an intriguing candidate because it is used in the baboon model of pertussis (9, 43 – 45). With the D420Sm1, we can have more accurate bacterial burden calculations while using a recent clinical isolate. This is crucial in evaluating the potential issues with our current aP vaccines. However, one caveat of using either D420 or D420Sm1 is that both strains retain a wild-type pertactin (PRN) gene which does not allow for evaluation of the lack of PRN antigen variable in regard to immunity.

In this study, we aimed to optimize the pertussis toxoid (PTd) mRNA antigen in a pertussis toxin (PT) challenge mouse model followed by an evaluation of the protection afforded by a novel 10-antigen mRNA-pertussis vaccine in an aerosol challenge model of *Bp* in rats. Coughing was measured with whole-body plethysmography, and cough counts for mRNA-vaccinated rats were comparable with the non-challenged group. Overall, the data presented here suggest that a novel mRNA-pertussis vaccine provides protection from pertussis manifestations and bacterial burden in the respiratory tract.

## RESULTS

### mRNA pertussis toxoid antigens to the A oligomer of PT alone are required for *in vivo* toxin neutralization

**Fig 1:**
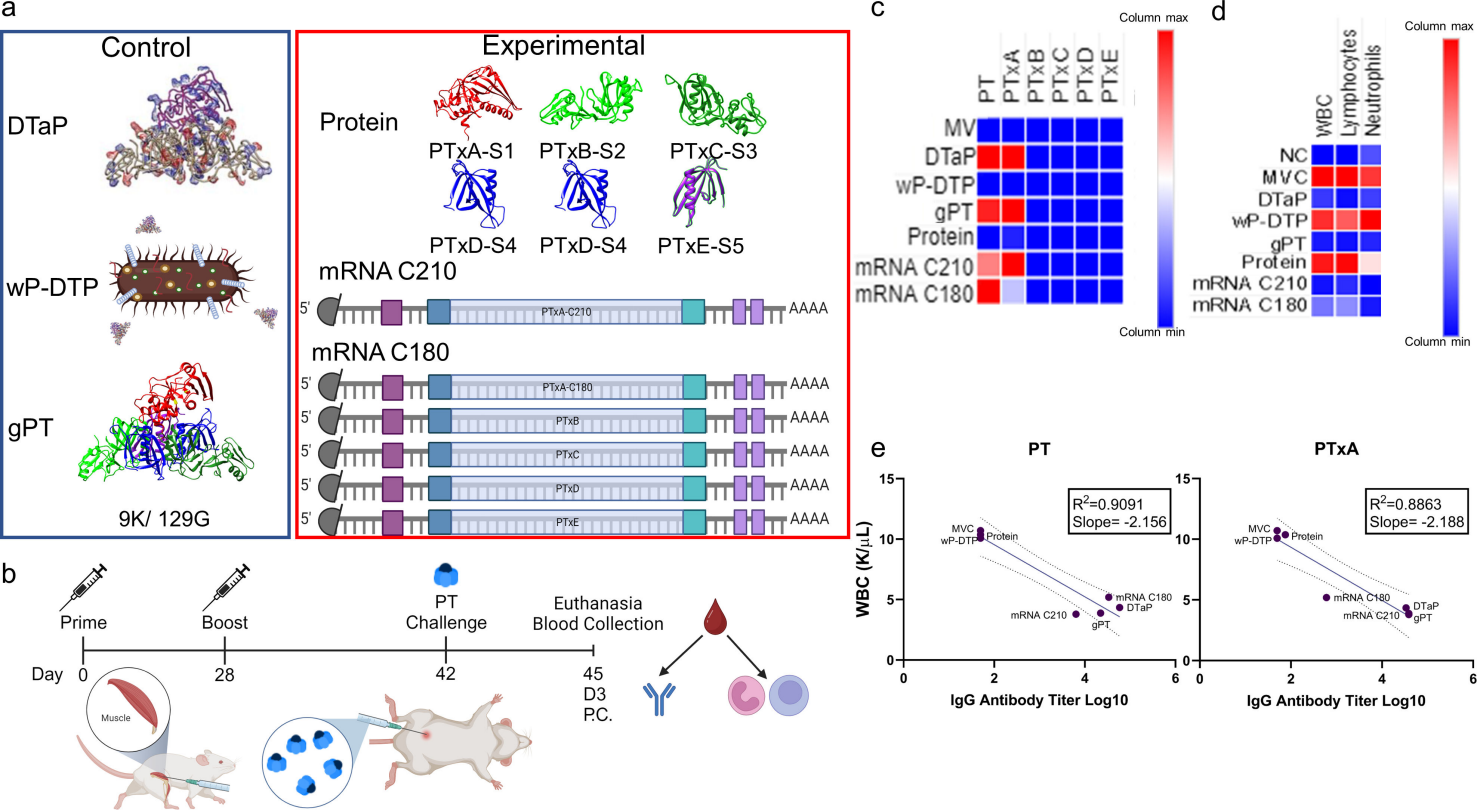
Comparison of mRNA antigens for pertussis toxin neutralization. Mice were immunized with either a control or experimental vaccine and challenged with purified PT to determine antibody-neutralizing capabilities. (**a**) A Chimera rendered image of detoxified PT in DTaP (Seubert et al. 2014) with lysine residues in red and arginine residues in blue as potential sites for structure deformity. Chimera was also used to render images of gPT showing the two mutations in yellow and the subunits of PT used in the experimental protein vaccine. (**a**) Graphical representation of the wP-DTP vaccine and mRNA antigens. The control vaccines are in the blue box, and the experimental vaccines are in the red box. (**b**) A schematic of the experimental timeline with intramuscular vaccination and intraperitoneal challenge with purified PT. Sera was collected 2 weeks post-boost from immunized mice and surveyed for IgG antibodies. (**c**) A heatmap of levels of specific IgG antibodies toward holotoxin and subunits of PT. (**d**) A heatmap of leukocyte values 3 days post-challenge with PT. (**e**) Correlation plots show an inverse relation between WBC counts and Log10 IgG antibody titers. WBC = white blood cell and MVC = mock-vaccinated and challenged.

We have observed that mRNA-pertussis vaccination is immunogenic, neutralizes toxins, and limits bacterial burden in mice (46). In those studies, we used an mRNA encoding only an A subunit of PT and did not evaluate if B oligomer subunits were necessary for protection. Previous studies used *in vitro* PT neutralization to determine antibody efficiency (47). However, here, we further evaluate mRNA-pertussis encoding for distinct PtxA antigens, one being transmembrane and one being soluble, as well as, B oligomer subunits to determine protection in an *in vivo* PT challenge model in mice as measured by reduced leukocytosis. With mRNA vaccine technology, it is possible to encode separate subunits as individual coding sequences. Since PT is made up of five subunits (PtxA, B, C, D, and E) with a ratio of 1:1:1:2:1, we aimed to evaluate if one, multiple, or all subunits are required for induction of neutralizing PT antibodies (Fig. 1a). Previously, we have shown that an mRNA antigen of PtxA (C180(48)) was sufficient to produce antibodies that protect against PT intoxication in mice (46). To properly evaluate mRNA and protein subunit vaccines, we encoded each PT subunit as a single mRNA construct, and we expressed and purified each PT subunit recombinantly from *Escherichia coli*. BALB/c mice were immunized with vaccine formulations described in Table 1. To establish baseline levels of PT antibodies induced by each commercial vaccine, mice were immunized with 1/20th human dose of DTP whole cell (wP), DTaP, and alum adjuvanted genetically detoxified Pertussis toxoid (gPT; Fig. 1a). We then immunized the experimental groups with recombinant individual PT subunit protein antigens, a pertussis mRNA C210, and a pertussis mRNA C180 (Fig. 1a). A graphical timeline illustrating this study is presented (Fig. 1b). We measured the amount of serum IgG antibodies to PT or recombinant antigens (PtxA, PtxB, PtxC, PtxD, and PtxE; Fig. 1c). We observed that all tested vaccines, including the commercially licensed vaccine controls, were not immunogenic for B oligomer subunits of PT (PtxB, C, D, and E), and we could not detect any antibodies to the B oligomer subunits (lower limit of detection; Fig. 1c). Not surprisingly, the wP-DTP did not induce an antibody response to PT or the subunits. This is because, during the production of whole-cell vaccine, the supernatant that contains the PT is discarded in a cell pelleting step, leaving antigens attached to the bacterium and only minimal supernatant antigens available in the vaccine. Interestingly, our protein subunit vaccine, which included all subunits of PT individually but not formed in a holotoxin, was also non-immunogenic and did not induce any detectable IgG antibodies to the whole PT or any of the subunits (Fig. 1c). Upon measuring IgG antibodies to whole PT, we found that DTaP (*P* = 0.001), gPT (*P* = 0.011), and mRNA C180 (*P* = 0.005) were significantly higher compared to the mock-vaccinated (MV) group (Fig. 1c). Unsurprisingly, when IgG antibody titers to PtxA were evaluated, we observed that both DTaP (*P* = 0.009) and gPT (*P* = 0.007) were significantly higher compared to the MV group. On the other hand, unlike antibodies bound to holotoxin, the mRNA C180 was not statistically different from the MV group; however, the mRNA C210 (*P* = 0.007) vaccinated group did induce statistically more PtxA-specific IgG antibodies than the MV group (Fig. 1c). This difference may be because certain epitopes of PtxA were presented in the single subunit form on an enzyme-linked immunosorbent assay (ELISA) plate which are hidden when the plate contains holotoxin. Additional statistical values are included in Table S1. We next wanted to evaluate the neutralization capacity of induced antibodies to PT in a mouse PT challenge model.

**TABLE 1:** Vaccine composition and detoxification^*a*^

Vaccine	Detoxification	Holotoxin	PTxA	PTxB	PTxC	PTxD	PTxE	Source
DTaP	Chemical	Yes	+	+	+	+	+	Infanrix
wP-DTP	Chemical	Yes	−	−	−	−	−	SII
gPT	Genetic	Yes	+	+	+	+	+	List Labs
Protein	None	No	+	+	+	+	+	Genscript
mRNA C210	Genetic	No	+	−	−	−	−	Moderna
mRNA C180	Genetic	No	+	+	+	+	+	Moderna

^*a*^ Vaccine composition for each group used in determining an optimal PT antigen. In a natural infection, pertussis toxin is secreted as a completed holotoxin, and the state of the pertussis toxoid in the vaccine is listed. If the vaccine contains a certain subunit of pertussis toxin, it is listed as “+,” and if it is absent, it is listed as “−.” The suppliers of each vaccine are listed. SII = Serum Institute of India.

**Fig 2:**
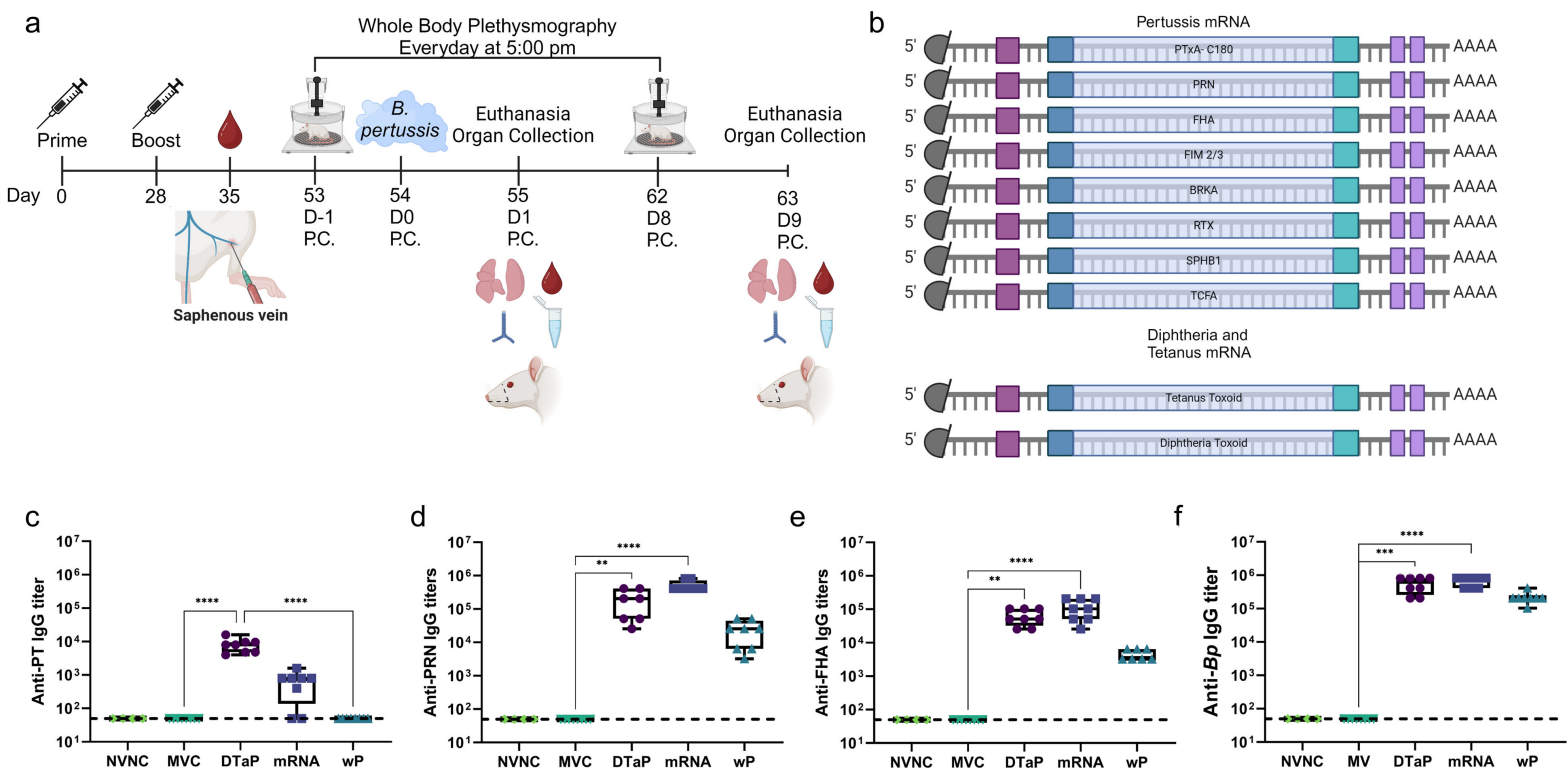
Experimental details of mRNA vaccination and immunogenicity in a rat model. (**a**) A graphical timeline of the study. The dashed lines on the rat graphic indicate the portion that was taken for the Nasal Associated Lymphoid Tissue (NALT) + Septum samples. (**b**) Graphical representation of the individual mRNAs that were included in the 10-antigen mRNA vaccine used in this study. Saphenous vein bleeds were conducted 1 week post-boost, and sera were collected and analyzed via ELISA for (**c**) PT-specific, (**d**) PRN-specific, (**e**) FHA-specific, and (**f**) whole *Bp*-specific IgG antibody titers. Data for c-f are presented as box and whisker plots showing all data points. A Kruskal-Wallis with Dunn’s multiple comparison post-hoc test was used to determine statistical significance against all groups. *P ≤ 0.05; **P ≤ 0.01; ***P ≤ 0.001; ****P ≤ 0.0001. Black dashed lines indicate the lower limit of detection. PT = pertussis toxin; FHA = filamentous hemagglutinin; NVNC = non-vaccinated non-challenged; wP = whole-cell pertussis; P.C. = post-challenge.

Mice challenged with *Bp* experience the effects of pertussis toxin; however, *Bp* expresses numerous toxins and virulence factors (49 – 52). Pertussis toxin injected directly into mice causes the systemic characteristic effects of PT (53). To evaluate only the neutralization of pertussis toxin, we used a direct PT challenge model where mice were administered 2 µg of active pertussis toxin by IP injection. Pilot studies revealed that leukocytosis was highest in mice 3 days post-challenge with PT. With that in mind, mice immunized with the control vaccines, PT protein subunits, or mRNA constructs were challenged with PT and evaluated at day 3 post-challenge (Fig. 1d). We expected that the vaccine groups that did not induce IgG titers to PT (wP-DTP, Protein subunit, and MV) would experience the highest levels of leukocytosis and lymphocytosis. When compared to the mock-vaccinated challenged (MVC) group, DTaP (*P* = 0.003), gPT (*P* = 0.0004), mRNA C210 (*P* = 0.0003), and mRNA C180 (*P* = 0.007) vaccinated mice had significantly reduced levels of white blood cells in the blood (Fig. 1d). This trend was similar in lymphocytes with a significant reduction in the DTaP (*P* = 0.005), gPT (*P* = 0.002), mRNA C180 (*P* = 0.045), and mRNA C210 (*P* = 0.005) groups compared to the MVC animals (Fig. 1d). Lastly, we investigated the number of neutrophils in the blood of PT-challenged animals. Again, we saw a similar trend with a significant reduction in the DTaP (*P* < 0.0001), gPT (*P* < 0.0001), mRNA C210 (*P* < 0.0001), and mRNA C180 (*P* < 0.0001) compared to the MVC animals (Fig. 1d). We next wanted to determine which mRNA S1 antigen to use in our future vaccines. The correlation between WBC levels and IgG antibody titers to PT and PTxA was plotted (Fig. 1e). Both plots had negative correlations which we expected, but the IgG titers to PT had a better *R* ^2^ value (0.9091 vs 0.8863). Overall, these data taken together suggested to us that we should move forward with the mRNA C180 antigen because it induced slightly higher levels of IgG antibodies to holotoxin than mRNA C210 and both had similar neutralization of PT as measured by leukocytosis and lymphocytosis. From here on, the PT antigen included will be mRNA C180, and as no B oligomer subunits are needed for protection, none were included in further mRNA vaccine formulation (Fig. 2b).

### Evaluation of an mRNA-pertussis vaccine in an aerosol challenge model using the coughing rat model of pertussis

We have previously shown in mice that mRNA DTP vaccines are immunogenic and are capable of protecting mice against *Bp* challenge (46). However, we aimed to extend upon those findings to evaluate protection against a major clinical symptom of pertussis which is the cough manifestation. IN administration of *Bp* can cause detectable coughing in rats (34, 35). However, we also aimed to improve this model to make it more relevant to natural exposure by switching from IN to aerosol challenge using a commercially available aerosol delivery chamber (36, 42). The overall graphical timeline of the study is presented in Fig. 2a. Rats were primed and boosted with the described vaccines and then bled post-boost/pre-challenge. After the aerosol challenge, rats were monitored by whole-body plethysmography. The individually coded pertussis mRNA included in the 10-antigen multivalent mRNA vaccine is pertussis toxoid subunit 1 (PTxA-C180), PRN, filamentous hemagglutinin (FHA), fimbria serotype 2 and 3 (FIM 2/3), Bordetella resistant to killing gene A (BrkA), repeats-in-toxin domain (RTX) of adenylate cyclase toxin, a subtilisin-like serine protease autotransporter (SphB1), and tracheal cytotoxin factor A (TCFA; Fig. 2b). The other two mRNAs included in the vaccine are Tetanus toxoid and Diphtheria toxoid to mimic what is found in current DTaP vaccines (Fig. 2b).

### The multivalent mRNA vaccine is immunogenic in Sprague-Dawley rats

Mice are known to respond highly to vaccine antigens, and therefore, testing a vaccine in another species is needed to determine if the vaccine is more likely to have potential as a candidate. We aimed to determine if the multivalent mRNA vaccine was immunogenic in Sprague-Dawley rats and to compare responses to DTaP and DTP vaccines. To do so, female Sprague-Dawley rats were primed at 4 weeks of age and boosted 28 days later with 1/10th human dose of DTaP, 1/10th human dose of wP-DTP, or 10 µg of mRNA-DTP. Blood was collected 1 week post-boost via saphenous vein blood draws, and IgG antibody titers to specific vaccine antigens were calculated (Fig. 2a). We observed that six of our eight rats immunized with mRNA induced measurable IgG antibodies toward PT post-boost (Fig. 2c). It should be noted that the PT antibody levels induced by the mRNA dose were lower than DTaP. We examined neutrophilia caused by PT in rats challenged with *Bp* strain D420Sm1 and observed that on day 1 post-challenge, the DTaP and mRNA vaccinated groups were able to limit neutrophilia to non-challenged levels indicated by the black dashed line (Fig. S1a). We also observed a similar number of neutrophils in the blood of MVC animals. This may be because the aerosol challenge model causes less severe disease compared to intranasal instillation of bacteria (36). There is another factor in which PT intoxication can originally subvert neutrophil recruitment during the early phase of infection and switch to induce neutrophilia later in the course of infection (54, 55). Interestingly, the wP vaccinated group had statistically higher neutrophil numbers compared to all groups on day 1 post-challenge (Fig. S1a). We hypothesize that this is due to the inflammatory immune response that wP immunization triggers (1, 6, 56). By day 9 post-challenge, we observed a statistically significant reduction for all vaccine groups compared to MVC in neutrophilia (Fig. S1b). This data would suggest that the mRNA vaccine was either able to induce PT-neutralizing IgG antibodies or reduced PT via another mechanism that limited neutrophilia in challenged animals. Next, we investigated IgG antibodies toward the adhesin PRN, and we observed that the mRNA group had comparable titers to the DTaP group and significantly more PRN-specific IgG antibodies than the wP immunized group (Fig. 2d). Antibodies toward another adhesin, FHA, followed the same trend with mRNA and DTaP vaccination inducing comparable FHA-specific IgG antibodies that was higher than the wP immunized group (Fig. 2e). More intriguing to us was when we measured IgG antibody titers toward the whole bacterium. In theory, most DTaP formulations have two antigens that are bacterial-bound proteins (PRN and FHA). The mRNA formulation has five bacterial-bound antigens (PRN, FHA, SphB1, BrkA, and Fim 2/3), and the wP formulation contains thousands of bacterial-bound antigens. However, the DTaP and mRNA both had higher IgG titers than the wP immunized groups, and both DTaP and mRNA induced significantly higher titers than the mock-vaccinated rats (Fig. 2f). Overall, these data suggest that mRNA vaccines are immunogenic in rats.

### A multivalent pertussis mRNA vaccine lessens bacterial burden comparable to licensed vaccines

**Fig 3:**
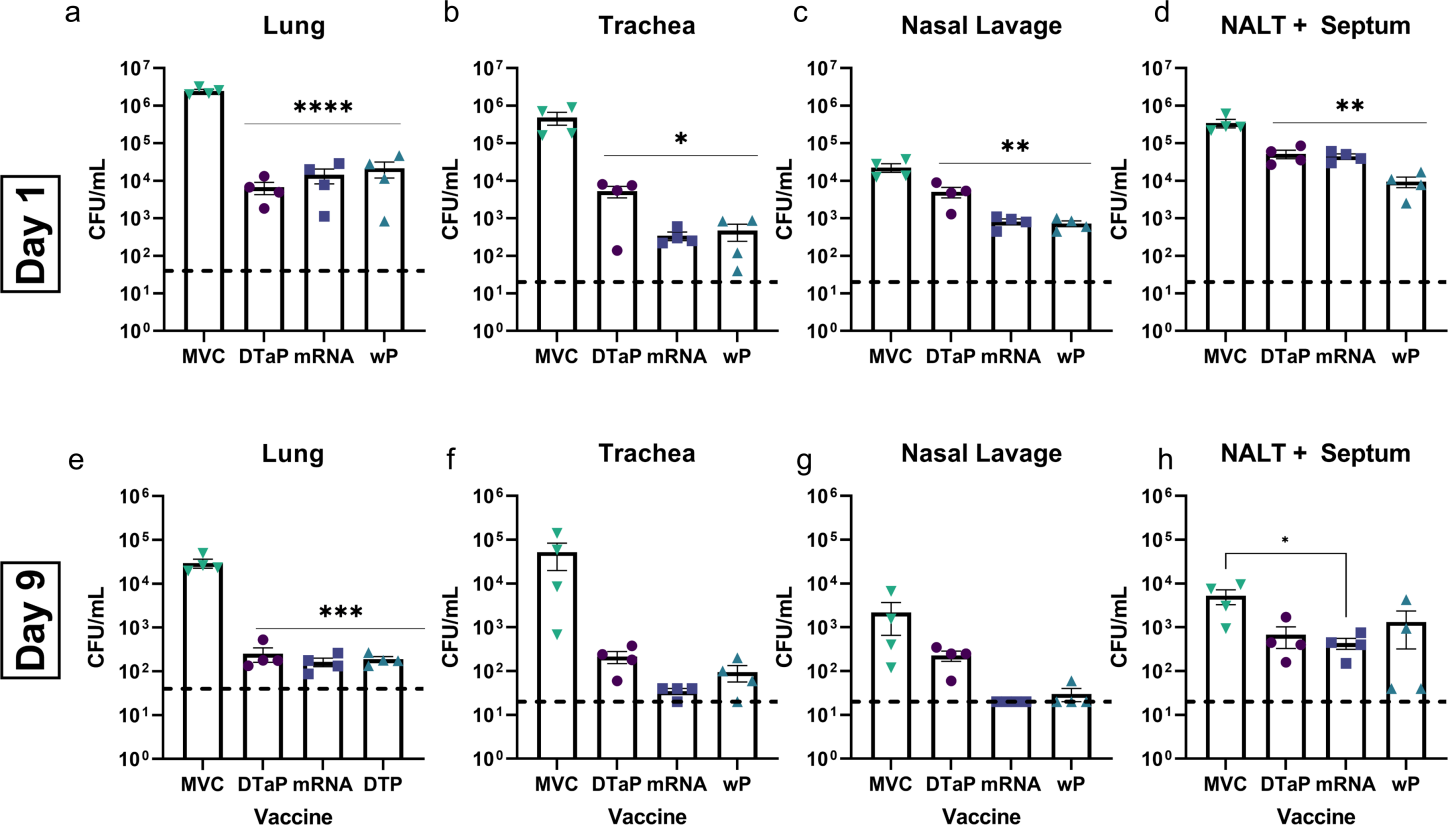
Bacterial burden is reduced in all vaccine groups. Bacterial burden was quantified in the (**a and e**) lung, (**b and f**) trachea, (**c and g**) nasal lavage, and (**d and h**) NALT + Septum of all challenged animals on days (**a−d**) 1 and (**e−h**) 9 post-challenge. Data presented on a log10 scale as mean ± SEM with all data points shown. Statistical significance was determined via a one-way analysis of variance(ANOVA) with Tukey’s post-hoc test against the MVC group. *P ≤ 0.05; **P ≤ 0.01; *** *P* ≤ 0.001; **** *P* ≤ 0.0001. Black dashed lines indicate the lower limit of detection.

We aimed to determine if the mRNA vaccine would limit bacterial burden in the coughing rat model. Previously, we have used IN challenge to deposit bacteria directly into the nares of animals; however, we elected to adapt our aerosol challenge model used in mice to rats for these experiments. The aerosol challenge model instills fewer bacteria than IN instillation. Furthermore, the lessened bacterial burden allows for better investigation of bacterial clearance due to vaccine-mediated immunity (36). In fact, by using the aerosol challenge model, we can see differences between the MVC groups and our vaccine groups when comparing bacterial burden in nasal lavage data; whereas in previous experiments, this difference is less obvious (data not shown). In this study, we did observe that the mRNA vaccine was able to significantly reduce bacterial burden compared to the MVC group comparable to both the DTaP and wP immunized groups on day 1 post-challenge (Fig. 3a through d). In the lung, we observed a >99% reduction for all the vaccinated animals compared to MVC (Fig. 3a). This same trend was observed in the trachea with an additional 93.5% reduction in bacterial burden for the mRNA group compared to DTaP immunized rats (Fig. 3b). There was also a significant reduction for all vaccine groups in the nasal lavage fluid compared to MVC with a further 83.8% and 85.7% reduction for the mRNA and wP, respectively, groups compared to the DTaP immunized animals (Fig. 3c). Lastly on day 1 post-challenge, we again observed a significant reduction in bacterial burden within the NALT (Nasal Associated Lymphoid Tissue) plus Septum homogenates for all vaccinated animals (Fig. 3d). We next assessed the protection induced by the vaccines to limit bacterial burden in the same organs and washes on day 9 post-challenge (Fig. 3e through h). At this time point, we observed that the MVC groups were able to reduce bacterial burden compared to their counterparts at day 1 by roughly two logs in the lungs, one log in the trachea, one log in the nasal lavage, and two logs in the NALT + Septum homogenates. However, we also observed an even further decrease in bacterial burden for vaccinated groups suggesting that all vaccines were able to confer protection. Again, we observed a >99% reduction in bacterial burden in the lungs of vaccinated animals compared to MVC (Fig. 3e). This reduction was statistically significant and comparable for all vaccine groups. We observed a similar reduction in the trachea of vaccinated animals, and although not significant, there was >99% reduction with all vaccine groups compared to MVC (Fig. 3f). Interestingly, there was an additional 83.7% and 55.8% reduction in the mRNA and wP immunized groups, respectively, compared to the DTaP group (Fig. 3f). The most important piece of data, in our opinion, was the ~99% reduction in the mRNA and wP vaccinated groups compared to MVC in bacterial burden found in the nasal lavage on day 9 post-challenge (Fig. 3g). We did not have detectable colonies in 4/4 and 3/4 of the mRNA and wP immunized rats, respectively, as indicated by the black dashed line (Fig. 3g). While it is certainly possible for bacteria to remain undetected in this tissue, the lower numbers of bacteria suggest that the mRNA vaccine was able to establish great bacterial clearance similar to that of wP vaccination. Finally, we observed a statistically significant reduction for only the mRNA group compared to MVC in the NALT + Septum (Fig. 3h). The mRNA group was also able to reduce bacterial burden by 35.4% and 67.2% compared to the DTaP and wP groups, respectively (Fig. 3h). These data taken together suggest that the mRNA vaccine induces bacterial clearance in the respiratory tissue of challenged rats comparable to, and in some cases better than, currently licensed pertussis vaccines.

### Coughing in challenged rats is significantly reduced with mRNA vaccination

**Fig 4:**
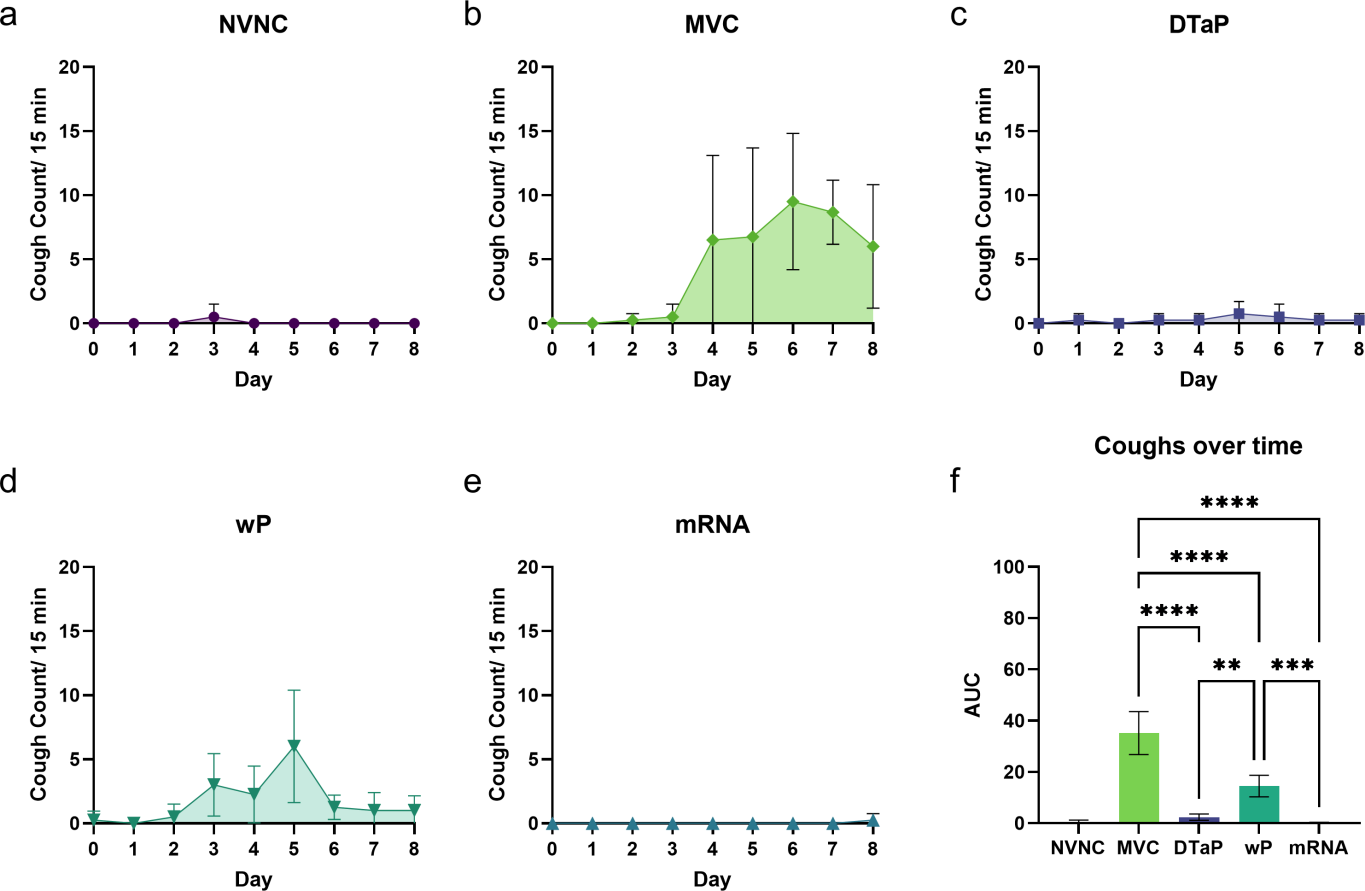
mRNA vaccination reduced *Bp*-induced coughing to non-challenged levels. Cough events from the rats were enumerated using whole-body plethysmography. Cough counts per 15 min were quantified for 8 days in groups of mice (**a**) not vaccinated and challenged, (**b**) mock vaccinated and challenge, or vaccinated with (**c**) DTaP, (**d**) wP, and (**e**) mRNA. (**f**) Area under the curve (AUC) analysis was performed for the variations in cough counts over the 8-day challenge window. Data for these figures are presented as mean ± SD showing all data points. A one-way ANOVA with Tukey’s post-hoc test was used to determine statistical significance between all groups. **P = 0.0018; ***P = 0.0003; ****P ≤ 0.0001.

The namesake disease manifestation of whooping cough is a violent cough which is not observed in mouse models of infection. However, a measurable coughing phenotype can be enumerated in the coughing rat model of pertussis (31). We have previously demonstrated that this model can be used to determine cough counts and other respiratory data related to *Bp* challenge in naïve and vaccinated rats (34, 35). In short, rats are placed within a commercially available chamber connected to a DSI Buxco Whole-Body Plethysmography device (Fig. S3b), and respiratory data are gathered at 1,700 every day from before the challenge until day 8 post-challenge (Fig. 2a). We observed that some animals began coughing on day 4 post-challenge (Fig. 4a; Fig. S2a). At that time point, the MVC group had the highest number of coughs with 2/4 of the animals exhibiting 10 or more coughs during the 15-min recording period (Fig. 4a and b). Both the mRNA vaccinated and non-vaccinated non-challenged (NVNC) groups had no detectable coughs during this period which was significantly reduced compared to the MVC group (Fig. 4a and f). The DTaP immunized rats also had significantly reduced coughing levels compared to the MVC group and had only one animal with a single cough on day 4 post-challenge (Fig. 4f). By day 5 post-challenge, we observed that the MVC and wP groups both had detectable coughs for all animals within the group, while mRNA and NVNC groups again had no coughs (Fig. 4a and e). The MVC rats increased their coughs on day 6 post-challenge, while all vaccine groups and the NVNC control groups had significantly reduced measured coughs (Fig. 4a and b). Similar to day 4, the NVNC and mRNA animals had no measurable coughs on day 6 (Fig. 4a and e). This trend was repeated on day 7 post-challenge (Fig. 4d), and it was not until the last day of the experiment that a single cough was detected in the mRNA-vaccinated group (Fig. S2b). The total coughs measured during the entire time course for each group were calculated, and not surprisingly, the MVC group had the highest number of coughs (Fig. 4f). Interestingly, the mRNA-vaccinated rats had a similar number of coughs as the NVNC groups and had fewer measured coughs than the DTaP vaccinated rats (Fig. 4f). In tandem, mRNA vaccinated rats had reduced bronchial restriction (measured by PenH) than the MVC rats on days 4 through eight post-challenge (Fig. S2b). Taken together, these data suggest that the mRNA vaccine does well at controlling coughing caused by a pertussis challenge and limits it to a number comparable to non-challenged animals.

## DISCUSSION

Antigen optimization in mRNA vaccines can provide an extra level of protection by selecting the best epitope regions to provide the highest degree of protection. In our first study, we established the optimal PT antigen to be included in the next study. It is known that PT is a major virulence factor of *Bp* (57). It is well appreciated that the native structure of gPT antigen induces superior neutralizing antibodies and higher overall amounts of antibodies post-immunization and boost than vaccines containing PTd (58 – 60). In conjunction, several studies have shown that monoclonal antibodies to certain epitopes of B oligomer subunits can protect mice from lethal cerebral challenge and others protect from aerosol *Bp* challenge in mice (61, 62). However, a previous study using recombinant S1 proteins determined that there was only one immunodominant epitope on the A subunit, and no immunodominant epitopes were found on recombinant S2−S5 proteins (63). With that in mind, we observed that neither aP, wP, nor mRNA vaccines were immunogenic for B oligomer subunits of PT (Fig. 1c). We observed that IgG antibodies to only the S1 (PtxA) portion of PT were needed to prevent leukocytosis in PT-challenged mice (Fig. 1d). We also observed that different portions of the PtxA protein encoded in mRNA can affect PT-specific IgG antibody production (Fig. 1c). Interestingly, the mRNA vaccines provided more PT-neutralizing antibodies than the recombinant protein vaccine. Both the mRNA and protein included individual subunit antigens, so the reasons for the poor neutralization in the protein vaccine are unclear. More studies will be needed to determine why the alum adjuvanted protein subunits were not immunogenic. Moving forward in this study, we determined that the C180 mRNA construct was the best PtxA construct evaluated (Fig. 1e); however, further work can be done to find the best potential epitopes to encode for optimal protection.

A key finding in this study was that mRNA pertussis vaccines are immunogenic in Sprague-Dawley rats (Fig. 2). To our knowledge, there are very few mRNA vaccines evaluated in rats. Previously, our work has determined that pertussis-specific mRNAs are immunogenic in BALB/c and C57BL/6j mice (46), but this work adds another murine model to this data. In our rat study, the mRNA produced comparable IgG titers specific PRN (Fig. 2d), FHA (Fig. 2e), and whole bacterium (Fig. 2f) compared to the humoral skewed DTaP control vaccine. There was also an increase in anti-PT IgG antibodies in the mRNA-DTP-vaccinated group compared to wP-vaccinated animals which do not contain a PT antigen (Fig. 2c). Although mRNA-DTP10 induced a slight reduction in anti-PT IgG titers compared to DTaP, there was still seroconversion in a majority of mRNA-DTP10 vaccinated rats. The two animals that did not seroconvert had anti-PRN, FHA, and whole-bacterium titers, suggesting to us that there was a technical issue in our assay and potential serum degradation upon multiple freeze/thaw cycles. The antibodies induced by vaccination can neutralize toxins, such as PT, and bind surface antigens of the bacteria to limit adhesion and promote complement killing (27, 64 – 67). The mRNA pertussis vaccine was able to induce neutralizing titers of PT as measured by leukocytosis and neutrophilia in both PT and aerosol-challenged animals (Fig. 1d; Fig. S1).

One issue with bacterial burden analyses is the presence of normal flora within the respiratory tract of mice and rats. Previously, we have used a streptomycin-resistant isolate (UT25Sm1) which was isolated in the 1970s (36, 68 – 70). However, genomic surveillance conducted by the Centers for Disease Control and Prevention in the US has shown that emerging clinical isolates differ greatly from original vaccine isolates in their genome and protein production (44, 71). The use of UT25Sm1 was efficient because it allowed streptomycin to restrict the growth of normal flora allowing for more accurate bacterial burden analysis. However, to better mimic a current *Bp* infection, an emerging clinical isolate should be used in challenge studies. In previous studies, we have been able to use cephalexin (mice) and ceftibuten (rats) to inhibit normal floral growth on agar plates because *Bp* is naturally resistant to these antibiotics (34, 72). However, many Gram-negative bacteria are naturally resistant which makes accurate bacterial burden analysis more challenging. Therefore, the use of an emerging clinical isolate with streptomycin resistance would allow for improved analysis with a relevant isolate.

One caveat to this study is that long-term immunity was not evaluated. The bacterial burden study showed that all vaccine groups were able to reduce the bacterial burden in respiratory tissues and lavages on days 1 and 9 post-challenge (Fig. 3). However, a study by Klein et al. suggested that protection afforded by aP vaccination wanes rapidly and is substantially gone by 2–3 years post-vaccination (73). The comparable reduction in bacterial burden by the DTaP-vaccinated rats may be an artifact of recent vaccination rather than a merit of the vaccine. Furthermore, a study by Pardi et al in 2018 showed that modified mRNA can induce a potent germinal center B cell and T follicular helper cell response compared to un-modified RNA (74). While our study did not evaluate a memory response from the novel mRNA-pertussis vaccination, we would speculate based on the previously mentioned study that the mRNA-pertussis vaccine would initiate a potent memory response, but further studies will be needed to conclude this. However, memory response aside, mRNA pertussis-vaccinated rats were able to clear all detectable bacteria from the nares by day 9 as all the animals in this group were at the lower limit of detection (Fig. 3g). This is a promising sign that mRNA pertussis vaccines may elicit a cell-mediated immune response that is more efficient at clearing bacteria. Further studies will need to be done to determine the immune cellular profile induced by this vaccine.

Most promising of all our data were the reduction in coughing observed in the mRNA pertussis vaccinated rats. Paroxysms are a hallmark disease manifestation of pertussis and the namesake of the disease (whooping cough). These coughs are debilitating and are so forceful that they have been known to crack rib bones (75). Although challenged rats do not manifest the hallmark paroxysmal cough, this model provides a coughing phenotype that can be enumerated and may be a valuable metric for protection from symptomatic pertussis in the future. Unsurprisingly, MVC rats showed the highest number of coughs during the recording period (Fig. 4f). DTaP vaccinations do well at limiting disease severity, and this was confirmed by the reduction in coughs recorded for this group during the study (Fig. 4f). More surprising was how well mRNA pertussis-vaccinated rats performed in this assay. Only one cough was recorded for one animal on the last day of measurement for the mRNA pertussis group (Fig. 4e and f). This is comparable to the non-vaccinated non-challenged group which were not challenged with *Bp*. This suggests that the mRNA pertussis vaccine was able to ameliorate cough occurrence and provided great protection to the vaccinated rats.

Another caveat to this study is that transmission was not evaluated in the coughing rat model. We expect that with the reduction in bacterial burden and coughs, the mRNA pertussis vaccine will limit transmission, unlike aP vaccination. However, another animal model of pertussis must be used to conduct these tests. The non-human primate model of pertussis (baboons) has proven invaluable in part because it allows for transmission studies (9, 43, 45). Warfel et al. showed that naïve and aP-vaccinated baboons challenged with *Bp* isolate D420 were able to transmit the bacteria to both co-housed and separated naïve baboons (9, 16). Our next step is to use this model with the mRNA pertussis vaccine to evaluate immunogenicity in non-human primates.

Other mRNA vaccines have been proven efficacious against bacteria such as *Borrelia burgdorferi* (Lyme Disease), *Yersinia pestis* (plague), and *Pseudomonas aeruginosa* (pneumonia) in pre-clinical trials (76 – 78). There have been two studies evaluating mRNA vaccines against viral pathogens including respiratory syncytial virus (RSV) and rabies virus in rats (79, 80). However, to our knowledge, the study presented here is the first pre-clinical study evaluating mRNA vaccines in rats against bacterial pathogens. The data presented here suggest that an mRNA pertussis vaccine is protective against an aerosol challenge in rats. Rats vaccinated with the experimental mRNA pertussis vaccine had reduced neutrophilia and coughing compared to MVC and wP-DTP vaccination. These rats also had reduced bacterial burden in the assayed respiratory tissue compared to MVC. Finally, mRNA pertussis vaccinated rats had comparable cough counts to the NVNC animals, suggesting that these rats were protected from the cough manifestation. Taken together, these data suggest that a next-generation mRNA pertussis vaccine is immunogenic and protects against pertussis and bacterial carriage in Sprague-Dawley rats.

## MATERIALS AND METHODS

### Vaccine composition for PT mRNA antigen optimization

DTaP (Infanrix; GSK) and wP (DTP; Serum Institute of India) were diluted to 1/20th of a single human dose. gPT was obtained through List Biological laboratories [cat#184 (50 µg)] and formulated to 1.25 µg per mouse dose to equal the amount of PT found in a 1/20th human dose of DTaP. Individual protein subunits of pertussis toxin were cloned, expressed, and purified by GenScript Biotech and combined in equimolar ratios to what is found in pertussis toxin (PtxA:B:C:D: E; 1:1:1:2:1), and the total amount used was 1.25 µg/mouse dose to equal the amount of PT found in a 1/20th human dose of DTaP. The gPT and protein subunit vaccines were adsorbed with Alhydrogel (Aluminum hydroxide; alum; InvivoGen; cat# vac-alu-250) by adding the equivalent amount of what would be found in a 1/20th human dose of DTaP, 3.125 µg, and mixed with end-over-end rotation for 1 h. The mRNA C210 and C180 LNP formulations (Moderna, Inc.) were diluted to 1 µg/antigen, and a total of 6 µg and 10 µg was used, respectively. Both mRNA C210 and C180 encode a truncated, genetically detoxified PtxA protein subunit (S1 subunit) of PT. The mRNA C210 encodes for a slightly larger protein and includes a transmembrane domain in place of the soluble factor in mRNA C180. All dilutions were done in sterile 1× endotoxin-free PBS (Millipore; cat# TMS-012-A). Five-week-old female BALB/c mice were primed with 50 µL of diluted DTaP, wP-DTP, gPT, protein subunit, mRNA C210, mRNA C180, or a PBS mock vaccine via the intramuscular route (50 µL) and boosted 28 days later with the same vaccine to align with the mRNA vaccination schedule. A table of vaccine formulations relating to PT has been included for clarity (Table 1).

### Pertussis toxin challenge model

Purified pertussis toxin [List Biological laboratories; cat#180 (50 µg)] was reconstituted in 500 µL of endotoxin-free 1× PBS to yield a concentration of 0.1 µg/ µL. Twenty microliters of this solution was combined with 180 µL of 1× endotoxin-free PBS to yield a challenge dose of 1 µg/100 µL. Vaccinated female BALB/c mice were challenged with 200 µL of the final pertussis toxin solution via intraperitoneal injection 2 weeks post-boost. Mice were then euthanized 3 days post-challenge via cardiac puncture, and the collected blood was placed in a microtainer K_2_EDTA tube (BD; cat# 365974). Fifty microliters of this blood was analyzed on a Procyte Dx Hematology Analyzer (IDEXX), and white blood cells, lymphocytes, and neutrophils were enumerated.

### Serological survey for PT antigen optimization

IgG antigen-specific antibodies in vaccinated and unvaccinated mice were measured via an ELISA. Plates were coated with 50 µL per well of either whole PT (List Biological Laboratories; cat#180) or an individual subunit of PT synthesized by GenScript Biotech (PTxA, B, C, D, and E) in a 1 µg/mL concentration. After overnight incubation at 4°C, plates were washed with 1× PBS-tween 20 and blocked with 5% non-fat dry milk (NFDM) in 1× PBS-tween 20 for 2 h at 37°C. Next, plates were washed, and a serial dilution of serum collected from post-boost submandibular bleeds was added to the plate. The last row was left without a sample to serve as a background control. The plates were incubated at 37°C for 2 h. After incubation, the plates were washed again, and 100 µL of a secondary goat anti-mouse IgG conjugated with alkaline phosphatase (AP; Southern Biotech; cat#1030–04) in a 1:2,000 dilution with 5% NFDM-1× PBS-Tween20 was added to each well. Plates were incubated at 37°C for 1 h to allow binding and then washed. One hundred microliters of *p*-nitrophenyl phosphate substrate was added to each well and developed in the dark at room temperature for 30 min. A BioTek Synergy H1 microplate reader was used to measure the colorimetric signal of the ELISA plates at A_405 nm_. A titer value was calculated as the last row with a positive reading defined as two times the average of the blank wells (background control). The limit of detection was set at 50 because our original dilution was 1:50, and any sample with no positive signal was set to this lower limit of detection.

### *B. pertussis* strain and growth conditions

*Bp* isolate D420 was provided by Drs. Michael Weigand and Maria Tondella at the Centers for Disease Control and Prevention in Atlanta, GA. This isolate was lab-adapted with a single SNP that induces streptomycin resistance to create the strain D420Sm1 (PRJNA998821). To do so, isolate D420 was passaged 3× on Bordet-Gengou (BG) agar with 100 µg/mL streptomycin. The resulting colonies were isolated, and the *rpsL* gene was sequenced by Sanger sequencing by Admera Health (South Plainfield, NJ). One isolate was confirmed to have the same SNP in the *rpsL* gene as streptomycin-resistant UT25Sm1. Illumina next-generation sequencing was done at Admera Health, and resulting reads were analyzed by the CDC to confirm no other major mutations existed between D420Sm1 and D420. D420Sm1 was cultured on BG agar (Remel, cat# R45232) supplemented with 15% defibrinated sheep’s blood (Hemostat Laboratories, cat# DSB500) and 100 µg/mL of Streptomycin (Toku-e, cat# S040-100g). Plates were incubated at 36°C for 48 h. After incubation, bacterial growth was transferred to Stainer-Scholte liquid Medium (SSM). Polyester swabs (Puritan, cat # 22–029-574) were used to transfer bacteria to SSM. Two milliliters of the liquid bacterial culture was pipetted into 18 mL of fresh SSM in a new 125 mL flask (Thermo Fisher Scientific, cat# FSB00125) and placed into a benchtop shaking incubator for 24 h at 36°C and 180 rotations per minute (RPM).

### Vaccine formulation and immunization for aerosol challenge study

DTaP (Infanrix, GSK) and wP (DTP, Serum Institute of India) vaccines were obtained from manufacturers and diluted to a 1/10th human dose with sterile 1× endotoxin-free PBS. The mRNA vaccine was obtained from Moderna, Inc. and contained single mRNA constructs of the following pertussis antigens: PtxA-C180, PRN, FHA, FIM serotype 2/3, BRKA, RTX, SphB1, and TCFA similar to as previously described (46). The mRNA vaccine also included two non-*Bp* antigens (TT, tetanus toxoid; DT, diphtheria toxoid) to mimic antigens found in DTaP and wP-DTP. These antigens were enveloped in Moderna’s proprietary lipid nanoparticle. A total of 10 µg of mRNA was used for each rat vaccination dose. Young, 4-week-old, female, Sprague Dawley rats were obtained from Charles River laboratories. Rats were randomly sorted by animal care technicians into two rats per cage. At 5 weeks of age, the rats were prime vaccinated with 100 µL of either DTaP, wP-DTP, mRNA, or 1× endotoxin-free PBS intramuscularly in the hind leg. The 1× endotoxin-free PBS served as the mock-vaccinated challenged control. Another group of rats was given neither vaccine nor challenged to serve as a baseline control. There were eight rats per vaccine group (four for the non-challenge group), and these were split into two groups of four. One group was euthanized on day 1 post-challenge, and the other was euthanized on day 9 post-challenge. Rats were then boosted with the same vaccine formulation 28 days after prime immunization and aerosol challenged 3 weeks post-boost.

### *B. pertussis* aerosol challenge

Rats were challenged with aerosolized *Bp* following the protocol by Bitzer et al. with some modifications detailed below (42). In short, *Bp* isolate D420Sm1 was grown as stated above. After incubation in SSM, the culture was diluted to an OD_600 nm_ of 0.240 ± 0.05 on a Beckman-Coulter UV-Vis Spectrophotometer which equates to 2–4 × 10^9^ CFU/mL. A total of eight rats were placed in the challenge chamber for each challenge based on the manufacturer’s instructions (Fig. S3a) (81). We performed four different challenges with each challenge including two rats from each group to compensate for small differences in the aerosol challenge. Four separate nebulizers were each filled with 5 mL of a 10^9^ CFU/mL dose of *Bp* for a total of 20 mL of dose per challenge. After a 5-min acclimation period, the dose is aerosolized within the sealed chamber over 10 min allowing the rats to naturally inhale the *Bp-*laced aerosols. After the challenge, rats were removed from the chamber and placed back in their corresponding cages.

### Serological survey of IgG antibody levels

At 1 week post-boost, blood was collected with capillary tubes via saphenous vein bleeds on the ipsilateral side of vaccination. Serum was obtained by centrifugation of capillary tubes at 15,000 × *g* for 3 min at 4°C. Sera was stored at −80°C until analysis. IgG and IgG subclass antibodies for specific antigens and whole bacterium were quantified through ELISAs. In short, high-binding 96-well plates (Pierce, ref#15041) were coated with 50 µL/well of the antigen of interest or D420Sm1 from a 1 µg/mL or ~10^9^ CFU/mL concentration, respectively. Coated plates were incubated overnight at 4°C and were dumped the next day. The plates were then washed three times with 1× PBS-tween 20 and 200 µL per well of 5% NFDM in 1× PBS-tween 20 was added to block non-specific binding. Plates were incubated for 2 h at 37°C and then removed, dumped, and washed three times with wash buffer. Upon analysis, samples were removed from the freezer and allowed to thaw on ice. A 1:50 dilution of each sample was done in 5% NFDM + 1× PBS-tween 20, and 100 µL was pipetted into the first row. A 1:1 serial dilution was performed in each subsequent row with the final row containing 50 µL of only the blocking solution to be used as the background control. Plates were incubated for 2 h at 37°C, then removed and washed three times with wash buffer. For antigen and whole-bacterium-specific ELISAs, a goat anti-rat IgG secondary antibody conjugated with AP (Southern Biotech; cat#3030–04) was diluted in a 1:2,000 dilution with a blocking buffer. For IgG subclass analysis, the following antibodies and dilutions were used: mouse anti-rat IgG1 (Southern Biotech; cat#3061–04) in a 1:2,000 dilution, mouse anti-rat IgG2a (Southern Biotech; cat#3065–04) in a 1:2,000 dilution, mouse anti-rat IgG2b (Southern Biotech; cat#3070–04) in a 1:4,000 dilution, and mouse anti-rat IgG2c (Southern Biotech; cat#3075–04) in a 1:2,000 dilution. One hundred microliter of the diluted secondary antibody mixture was added to each well, and the plates were incubated at 37°C for 1 h. After, plates were dumped and washed three times and had 100 µL of *p*-nitrophenyl phosphate substrate added per well. Plates were allowed to develop for 30 min in the dark at room temp and subsequently read at A_405nm_ on a BioTek Synergy H1 microplate reader to measure the colorimetric signal. The titer value was calculated as the last row with a positive reading defined as two times the average of the background control wells. Based on the initial 1:50 dilution, a titer value of 50 was used as the lower limit of detection.

### Bacterial burden of respiratory tissues

On days 1 and 9 post-challenge, rats were euthanized with pentobarbital, and a cardiac puncture was performed. The lungs were excised and placed in a GentleMACS C tube with 2 mL of 1× PBS. The trachea was excised and placed in a 15 mL culture tube containing 2 mL of 1× PBS. Nasal lavage was performed with 2 mL of 1× PBS and collected in a 15 mL conical tube. The rat head was skinned, and the lower jaw was removed. A scalpel was used to remove the NALT from the roof of the mouth and placed in a 15 mL culture tube with 2 mL of 1× PBS. The septum was removed by cutting just above the front teeth, and a second cut was made on a 45° angle toward the eyes. Once cut, the septum was placed in the same tube as the NALT. The lungs were homogenized with a GentleMACS Octo tissue dissociator on setting m_Lung_02_01. The trachea and NALT + Septum were homogenized with a Polytron benchtop homogenizer. The NALT + Septum samples were filtered through a 70 µm mesh filter before bacterial burden analysis. Samples were serially diluted 1:10 in 1× PBS, and four technical replicates were plated for each dilution onto BG agar supplemented with 100 µg/mL streptomycin and 15% defibrinated sheep’s blood. Plates were incubated for 72 h at 37°C before CFUs were counted and bacterial burden determined per tissue.

### Hematology analysis of post-challenge blood

Blood was collected via cardiac puncture, and 250 µL was placed in a K_2_EDTA microtainer tube and placed on ice. For analysis, 50 µL of blood was placed in a 1.5 mL Eppendorf tube and placed in an IDEXX Procyte Dx Hematology analyzer to determine blood levels on neutrophils.

### Respiratory distress and coughing analysis in rats

Whole-body plethysmography was performed similarly to previous publications (34, 35). Briefly, beginning at 5:00 pm from the day before the challenge (baseline) to day 8 post-challenge, rats were placed in designated chambers within a Buxco FinePointe Whole-Body Plethysmography system (WBP; DSI; Fig. S3b). Once placed in their chambers, rats were given a 5-min acclimation period before a 15-min recording period. During the recording period, a screen pneumotach allowed for inflow and outflow air to be measured. Cough events were determined using FinePointe software (DSI, v2.7.0.11788) by large box flow volume changes and patented fuzzy logic criteria (Fig. S3c) (82). In the case of a multi-cough event, each cough was counted individually.

### Statistical analysis

Prism version 9.0 software (GraphPad) was used for all statistical analyses. A one-way ANOVA followed by Tukey’s post hoc test was used for all comparisons between three or more groups with parametrically distributed data. A Kruskal-Wallis followed by a Dunn’s multiple comparison post hoc test was used for nonparametric data.

## Data Availability

Data for all figures are available upon reasonable request to the corresponding author.

## SUPPLEMENTAL MATERIAL

Figure S1 iai.00520-23-s0001.tif mRNA vaccination in rats reduces neutrophilia.

Figure S2 iai.00520-23-s0002.tif Respiratory data.

Figure S3 iai.00520-23-s0003.tif Graphical illustration of aerosol challenge and whole-body plethysmography.

Table S1 iai.00520-23-s0004.tif Table of p-values for each vaccine group to MVC for Fig. 1.

ASM does not own the copyrights to Supplemental Material that may be linked to, or accessed through, an article. The authors have granted ASM a non-exclusive, world-wide license to publish the Supplemental Material files. Please contact the corresponding author directly for reuse.
