# Supplementary figures and images for: Immunization with an mRNA DTP vaccine protects against pertussis in rats
Source: Infect Immun. 2024 Jul 17;92(8):e00520-23. doi: 10.1128/iai.00520-23 (PMC11320933; doi:10.1128/iai.00520-23)

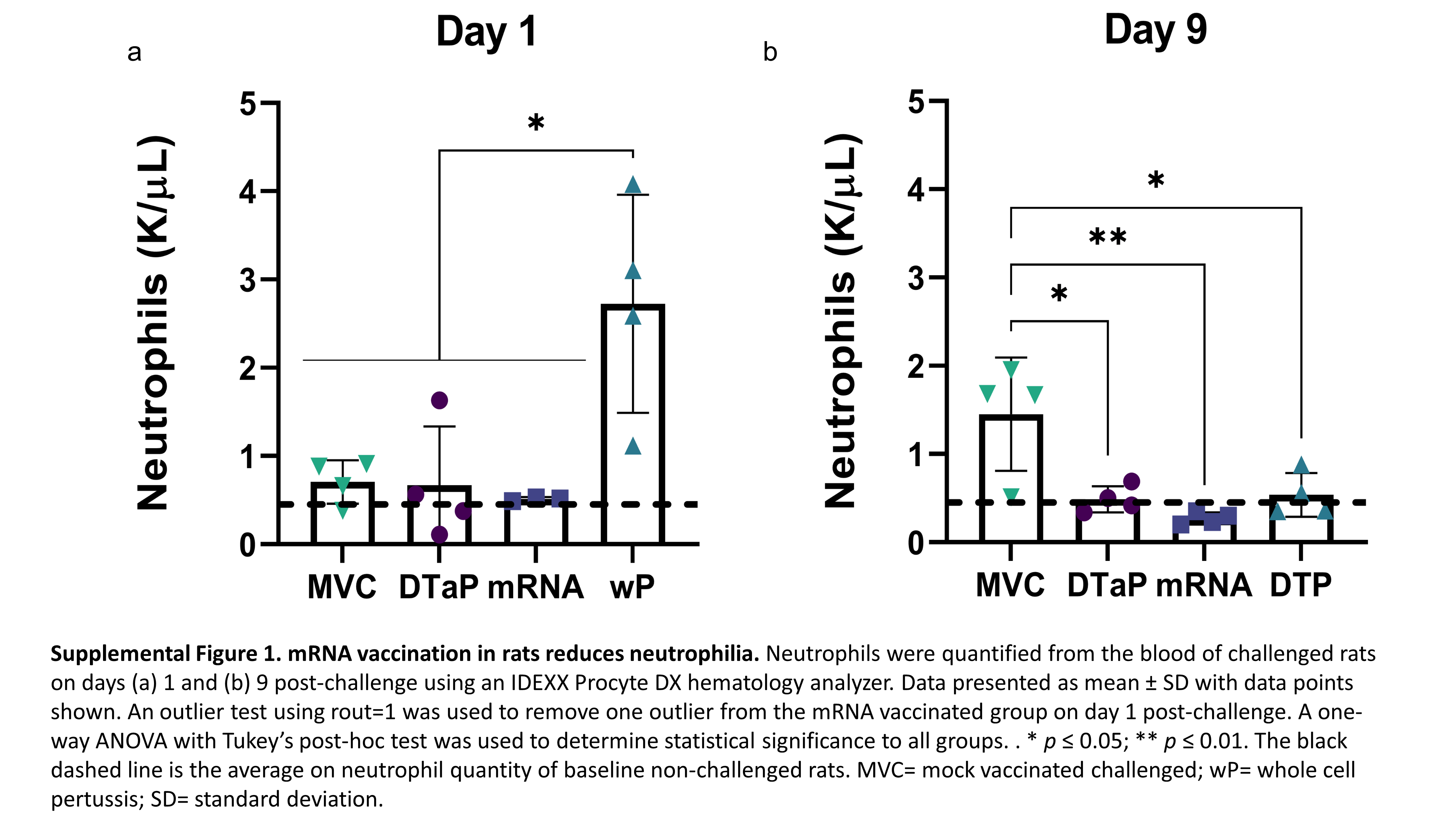

Supplement: Figure S1 — mRNA vaccination in rats reduces neutrophilia. [file iai.00520-23-s0001.tif]

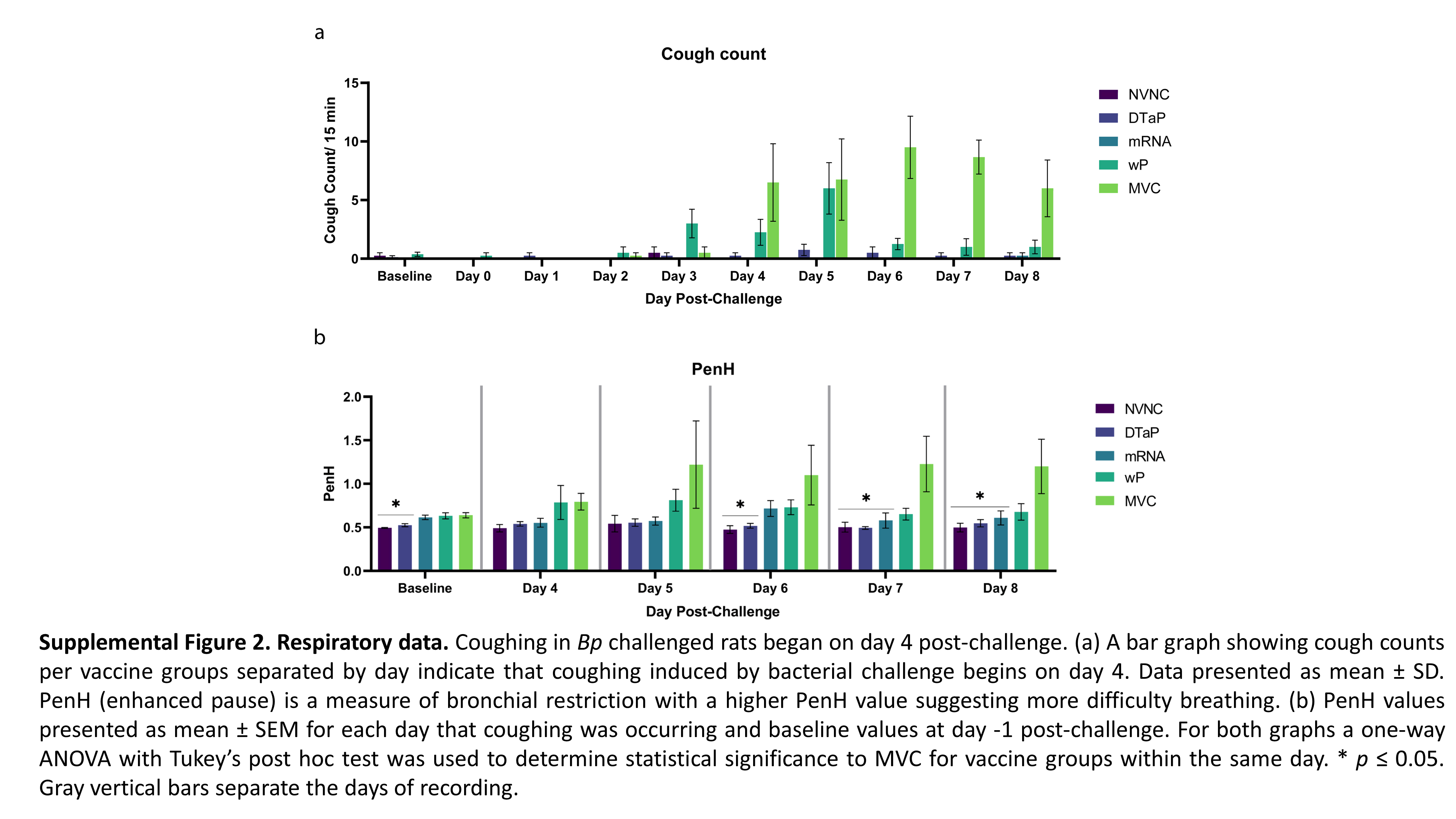

Supplement: Figure S2 — Respiratory data. [file iai.00520-23-s0002.tif]

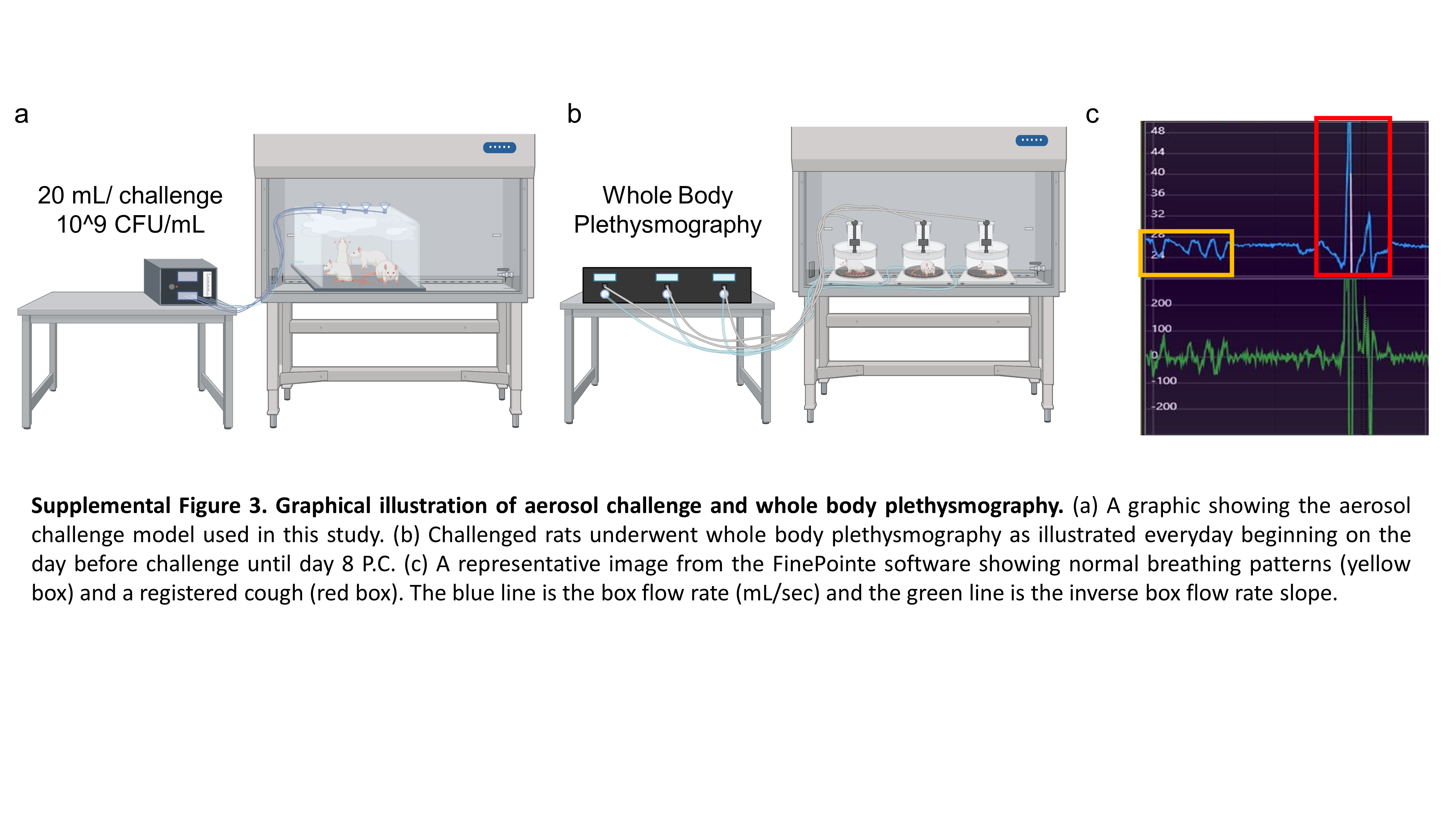

Supplement: Figure S3 — Graphical illustration of aerosol challenge and whole-body plethysmography. [file iai.00520-23-s0003.tif]

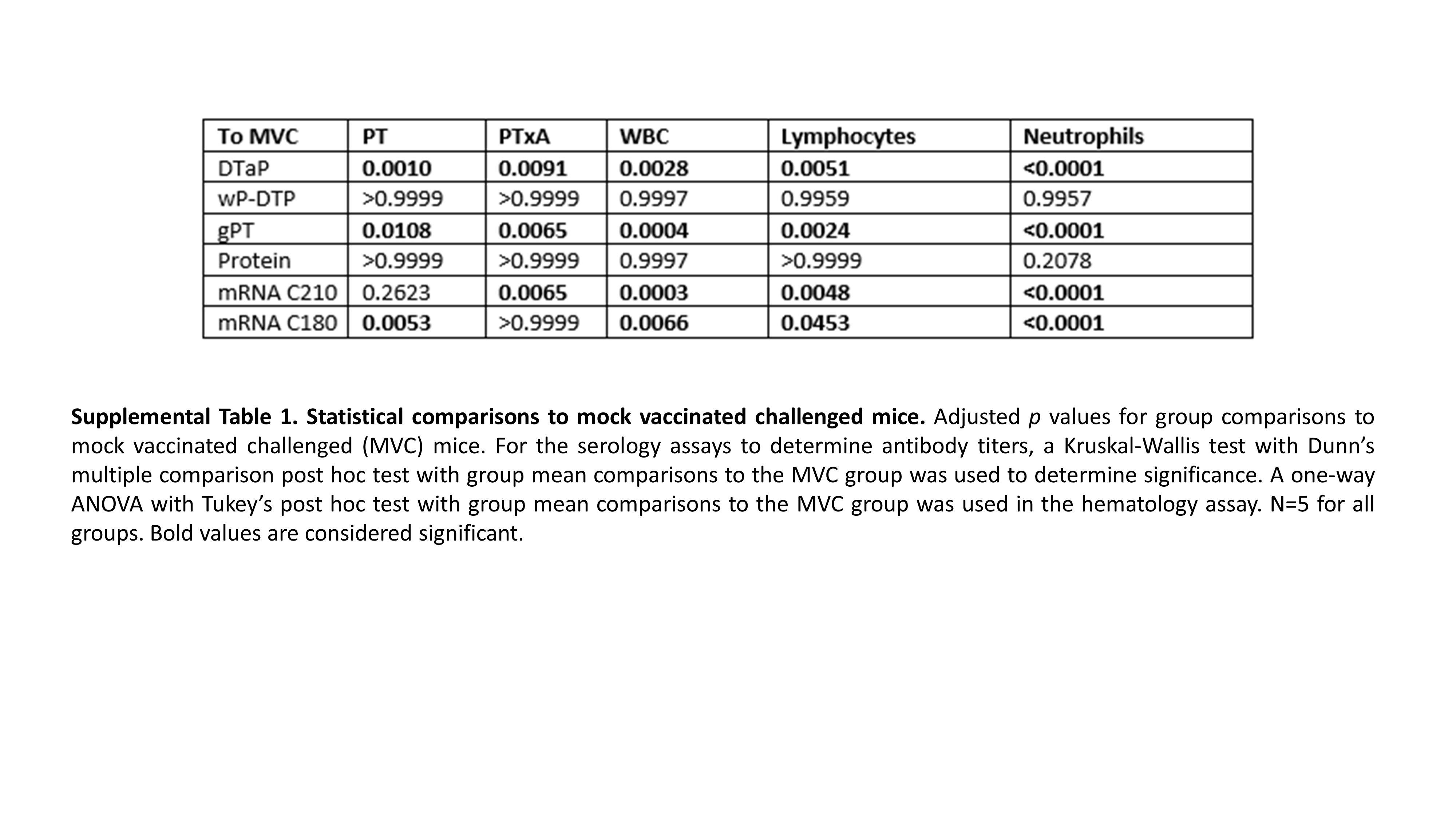

Supplement: Table S1 — Table of p-values for each vaccine group to MVC for Fig. 1. [file iai.00520-23-s0004.tif]
